# Advanced HCC Patient Benefit From Neoantigen Reactive T Cells Based Immunotherapy: A Case Report

**DOI:** 10.3389/fimmu.2021.685126

**Published:** 2021-07-13

**Authors:** Chenxi Liu, Jie Shao, Yanbing Dong, Qiuping Xu, Zhengyun Zou, Fangjun Chen, Jing Yan, Juan Liu, Shuangshuang Li, Baorui Liu, Jie Shen

**Affiliations:** Comprehensive Cancer Centre of Drum Tower Hospital, Medical School of Nanjing University, Clinical Cancer Institute of Nanjing University, Nanjing, China

**Keywords:** hepatocellular carcinoma, neoantigen reactive T cells, immunotherapy, tomotherapy, benefit

## Abstract

Advanced hepatocellular carcinoma (HCC) is a highly lethal disease, mainly due to the late stage at diagnosis and its rapid progression. Although patients with advanced HCC can choose targeted therapy or chemotherapy, overall, the treatment response rate is extremely low and the average survival time is one year more or less. But the application of immunotherapy have led to a paradigm shift in the treatment of HCC,such as TILs (tumor infiltrating lymphocytes),Checkpoint blockade (immune Checkpoint blockade), CAR-T(chimeric antigen receptor T cells) and TCR-T (engineered t-cell receptor T cells). And recent data indicate neoantigens generated when tumors mutate are the main target of tumor-specific TILs, and they are also the main antigens mediating tumor regression in TILs treatment. Moreover, numerous evidences have revealed that radiotherapy lead to massive release of tumor antigens, which may increase the effectiveness of immunotherapy. Based on the above theory, we used neoantigen reactive T cells combined with tomotherapy to treat a patient with advanced HCC (Clinical Trial Study Registration Number: NCT03199807), who reached a long time progress free survival.

## Introduction

In China, hepatocellular carcinoma is among the top five cancer types that have high morbidity and mortality (1). Approximately 80% of the HCC patients were initially diagnosed in an advanced or metastatic stage. Surgery is the first treatment for HCC patients, but not many patients are eligible for surgery, and the 5-year risk of recurrence after hepatectomy is as high as 50%-70% (2, 3). Interventional therapy and radiotherapy are mostly used as auxiliary treatment methods. In terms of targeted therapy or chemotherapy, the average survival time of patients receiving this treatment is one year more or less, and the remission rate is 2%-18.8% (4–7). Although the use of PD-1 can effectively prolong the survival of patients, the response rate of single-drug use is no more than 16-20%, this suggests that the use of single drugs needs to screen effective populations. In summary, looking for more effective immunotherapy may become a new breakthrough in the field of current HCC treatment.

In a clinical trial of metastatic melanoma, Dr. Rosenberg and his colleagues, who worked at NCI in the United States, confirmed that TILs back-transfusion combined with non-myeloablative chemotherapy or radiotherapy could achieve a clinical remission rate of 40%-72%, and nearly 40% of patients with complete remission (CR) showed no recurrence for more than 7 years (8). The above data indicate that TILs may bring new hope for the treatment of HCC, and the neoantigens that are the main targets of TILs, with its unique advantages, it has become a hot field in today’s anti-tumor research.

Neoantigens are a class of antigens derived from normal human genomes. Antigens produced by mutant proteins and oncoviruses are integrated into human genome. They have high affinity with TCR and strong immunogenicity. Compared with the traditional tumor-associated antigen (TAA), neoantigen is not expressed by normal tissues; and thereby, it does not cause central immune tolerance or autoimmune diseases (9). In addition, in 2015, Schumacher TN et al. conducted a tumor mutation spectrum analysis on several common solid tumor types, and showed that the mutation frequency of liver cancer was among the highest (10), and radiotherapy lead to massive release of tumor antigens (11). Based on the above principles, neoantigen reactive T cells combined with tomotherapy have been considered as a promising therapy for advanced cancer. In our previous study, we have combined TCGA data and the NGS sequencing results of the liver cancer samples from our center to screen out the genes with high-frequency mutations (12). Based on the common HLA genotyping (HLA-a*02:01, HLA-a*02:03, HLA-a*02:06, HLA-a*11:01, HLA-a*24:02) in Chinese population, 29 epigenetic peptides were selected and validated the antigenicity and other related parameters of the library (12), and finally we further conducted the clinical study that combined neoantigen reactive T cells with tomotherapy (Clinical Trial Study Registration Number: NCT03199807). Here, we reported a case that had significantly beneficial results from this therapy. Written informed consent was obtained from the individual for the publication of any potentially identifiable images or data included in this article.

## Case Presentation

The patient was male, 75-year-old, with a history of chronic hepatitis B. Partial hepatectomy was performed after the diagnosis of primary liver cancer in Apr-2016. In Oct-2016,abdominal Computed Tomography (CT) re-examination revealed multiple enhanced nodules in the liver, and the possibility of metastasis was considered. Therefore, this patient underwent 3 Transarterial Chemoembolization (TACE) operations.

Unfortunately, the abdominal Magnetic Resonance Imaging (MRI) re-examination in Feb-2017 revealed multiple metastatic liver lesions with enlarged hilar lymph nodes.

The patient strongly requested to participate in our clinical trial of tomotherapy combined with Neoantigen Reactive T (NRT) Cells based immunotherapy. In March 2017, this patient received tomotherapy for local lesions in the right anterior lobe of the liver; the cumulative dose was: Planning Target Volume (PTV): 50Gy/10f. After tomotherapy, the patient received personalized NRT immune cells reinfusion combined with IL-2(400WU/d, civ, 5 days in total) and GM-CSF cytokine (150ug/d, ih, 5 days in total) therapy in March 2017, with one month per cycle and a total of four cycles. According to the patient gene expression profile, the neo-antigen was chosen as: (HLA-a*11:01): KRAS-(G12A) p07-16: VVVGAAGVGK; KRAS-(G13D) p07-16: VVVGAGDVGK; PIK3CA-(H1047L) p1046-1054: ALHGGWTTK; IDH1(R132H)p123-142: GWVKPIIIGHHAYGDQYRAT.

**Figure 1:**
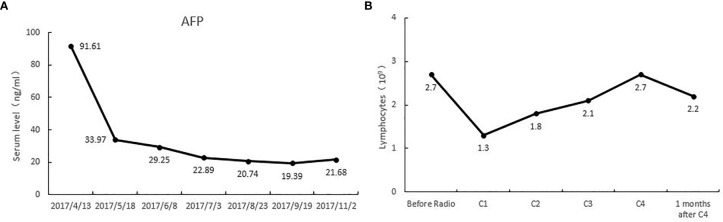
**(A)** alpha fetoprotein (AFP) levels of this patient during the treatment of tomotherapy combined with Neoantigen Reactive T Cells. **(B)** The lymphocyte levels of this patient during the treatment of tomotherapy combined with Neoantigen Reactive T Cells.

This patient was PD-L1(+), and TMB-H. The re-examination after 2 cycles of treatment showed that the lesion on the right anterior lobe of the liver became smaller, and the number of nodules in the more enhanced focus was reduced. The tumor markers were significantly down-regulated, and the changes in HCC specific tumor marker AFP were listed in **Figure 1A**. At the same time, the lymph number gradually increased, and the changes in lymphocytes after per cycle of NRT treatment were shown in **Figure 1B**. After four cycle of NRT, the overall efficacy evaluation was SD, and only regular examination was continued. The amount of T cells reinfusion per cycle and the amount of NRT immune cells detected *in-vivo* were listed in **Table 1**. Except for the fever during the treatment (the highest temperature was 38.3°C, and it was relieved after Paracetamol), there was no other discomfort or obvious liver toxicity.

**Table 1:** The amount of T cells reinfusion per cycle and the amount of NRT immune cells detected *in-vivo*.

	Number of NRT (10^10^)	CD3^+^CD4^+^CD137^+^ (10^8^)	CD3^+^CD8^+^CD137^+^ (10^8^)
First cycle	1.03	0.62 (0.6%)	0.82 (0.8%)
Second cycle	2.04	1.02 (0.5%)	3.47 (1.7%)
Third cycle	0.43	1.08 (2.5%)	0.52 (1.2%)
Fourth cycle	1.12	0.78 (0.7%)	2.13 (1.9%)

**Figure 2:**
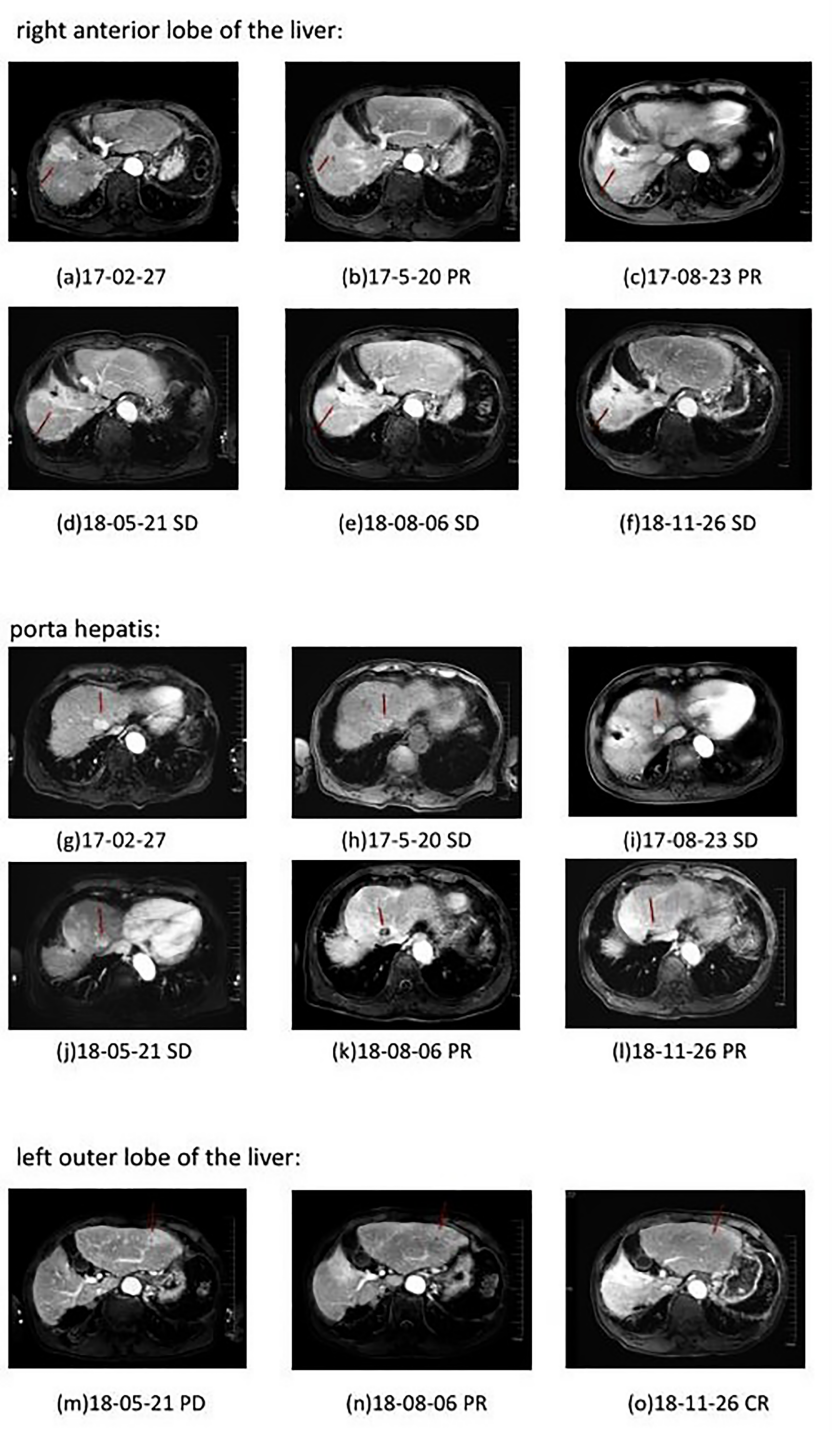
Plain and enhanced MRI of lesions in different parts of patient in different periods. a–f: right anterior lobe of the liver; g–l: porta hepatis; m–o: left outer lobe of the liver. Efficacy evaluation: PD, Disease progression; SD, Stable disease; PR, Partial response; CR, Complete response.

**Figure 3:**
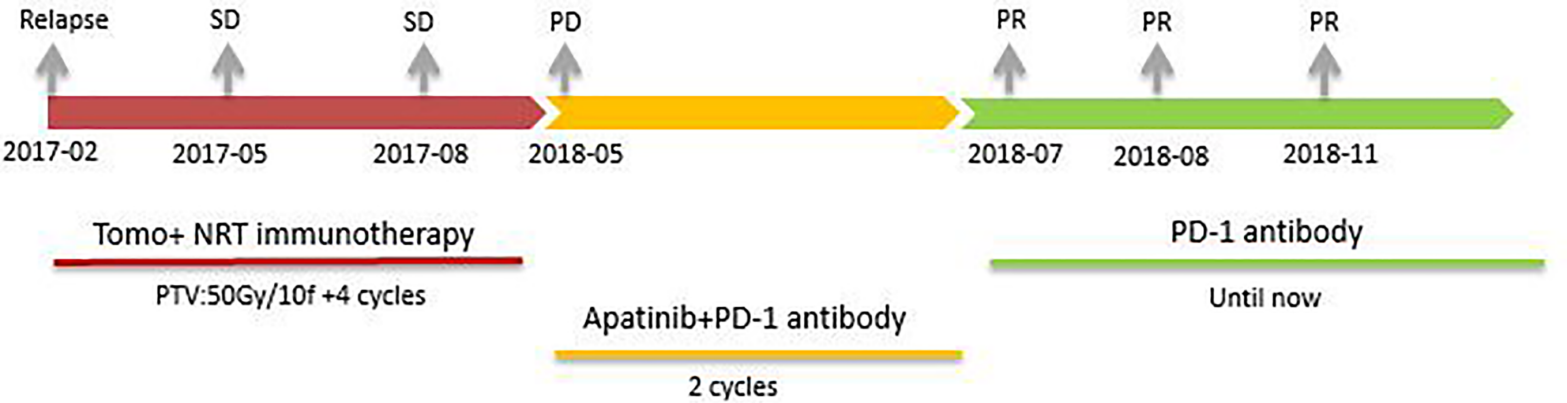
The patient received different treatments at different times. Firstly, tomotherapy combined with Neoantigen Reactive T Cells based immunotherapy(2017-02 to 2017-08, SD). Secondly, PD-1 antibody combined with apatinib(2018-05 to 2018-07, PR). Finally, PD-1 antibody single-drug maintenance treatment (2018-07 until 2020-06, PR in porta hepatis and CR in left outer lobe).

10 months after the radiotherapy combined with personalized cellular immunotherapy, a review of the abdominal MRI on May 2018 showed that the left outer lobe of the liver appeared, which was considered as disease progression. After full informed consent, the patient was enrolled in the RESCUE project (13)with combined PD-1 antibody and apatinib. From 2018-6, the patient received intravenous injection of PD-1 antibody(SHR-1210,200mg/q2w,iv, two times as a cycle) and apatinib (250mg/d,po). After 2 cycles of PD-1 antibody treatment, the re-examination showed that the intrahepatic mass of porta hepatis and left outer lobe were significantly smaller than before, with the overall efficacy evaluation as partial response (PR). However, due to the hand-foot syndrome side-effect of apatinib, which seriously affected the daily activities of patient, the patient stopped taking apatinib from 2018-7. Therefore, since then, PD-1 antibody single-drug treatment was performed till 2020-6, lasting for a total of 2 years. The patient has been in a fairly good condition. The regular reviews of the tumor showed gradually shrinking of lesion in porta hepatis, and complete response (CR) of intrahepatic lesions in left outer lobe. The patient’s imaging changes were shown in **Figure 2**. The target lesion changes at different time points during and after the treatment were listed in **Table 2**.In order to intuitively reflect the patient’s prognosis and clinical curative effect, we listed the entire treatment process in **Figure 3**.

**Table 2:** The target lesion changes at different time points during and after the treatment.

	2017-2 (mm)	2017-8 (mm)	2018-5 (mm)	2018-8 (mm)	2018-11 (mm)	2020-07 (mm)	2021-3 (mm)
right anterior lobe	33.60	22.93	18.45	18.65	18.60	18.62	18.69
porta hepatis	21.48	18.35	19.08	7.71	9.25	6.21	6.21
left outer lobe	none	none	18.00	7.11	0	0	0

## Discussion

This is a case of comprehensive treatment with obvious benefits. In the first stage, the patient benefited from tomotherapy combined with Neoantigen Reactive T Cells. The lesion with radiotherapy was significantly reduced, and the remaining lesions were stable. In the second stage, new lesion appeared on the left lobe, and the patient benefited from the treatment of apatinib combined with PD-1 antibody. At the final stage, PD-1 antibody maintenance treatment was going on after the abandonment of apatinib because of the serious side effects, this patient continued to get benefit from treatment. From the genetic test, this patient is positive for TMB-H and PD-L1, which may be a potential predictor of his benefit from immunotherapy. At the same time, this patient has mutations in KRAS and IDH1, which are frequent mutation sites in HCC, providing a genetic basis for neoantigen immunotherapy. The patient’s early cell therapy provides a material basis for the subsequent PD-1 antibody to a certain extent.

In this comprehensive treatment, it reflects the synergy of radiotherapy and immunotherapy. In recent years, the synergistic effect of radiation and immunotherapy has been reported widely. Radiation can cause tumor to release tumor antigens, transforming cold tumors into hot tumors. And these changes in immune microenvironment can attract T cells to attack the tumor site, thus synergizing the effects of radiation and immune response (14–16). In addition, radiotherapy can lead to a significant decrease in lymphocyte count, which possibly eliminates the inhibitory or dysfunctional T cells. We also observed this change in our study. The number of lymphocytes in patients declined significantly after radiotherapy. But after we transfused the T cells back into patients, the number of lymphocytes gradually increased and played a synergistic anti-tumor role.

In this comprehensive treatment, it also reflects the synergy of anti-angiogenesis and immunotherapy. Studies have shown that the basic principle of anti-angiogenic drugs combined with immunotherapy is that anti-angiogenic drugs can inhibit the occurrence of angiogenesis and immune escape in the tumor microenvironment, at the same time, this effect can enhance the body’s immune response to achieve anti-tumor effects (17, 18). The Keynote524 study also shows that when the two drugs are used in combination, the objective response rate(ORR) of patients with advanced HCC can reach 36%, progression-free survival (PFS) reaches 8.6 months, and overall survival (OS) reaches 22 months (19). We also observed a significant reduction of the lesion in the study. After the patient’s left outer lobe of the liver was treated with anti-angiogenesis combined immunotherapy, the lesion was significantly reduced, and the efficacy was evaluated as PR.

Overall, this comprehensive treatment model is safe and well tolerated. Except for hand-foot syndrome caused by apatinib, there is no other special discomfort. But it brings significant survival benefits to patients.

## Data Availability Statement

The datasets presented in this study can be found in online repositories. The names of the repository/repositories and accession number(s) can be found in the article/supplementary material.

## Ethics Statement

The studies involving human participants were reviewed and approved by Medical Ethics Committee of Drum Tower Hospital Affiliated to Nanjing University Medical School. The patients/participants provided their written informed consent to participate in this study.

## Author Contributions

JShe and BL designed the clinical trial. CL, JShe and QX composed the manuscript and provided figures. CL, JSha, YD, QX, JY, JL, BL and JShe did the work of the acquisition, analysis and interpretation of the data. JShe, YD, ZZ, FC and SL revised the manuscript critically for important intellectual content, and agreement to be accountable for all aspects of the work, in ensuring that questions related to the accuracy or integrity of any part of the work are appropriately investigated and resolved. All authors ontributed to the article and approved the submitted version.

## Funding

This study was supported by National Natural Science Foundation of China (No. 81902914); Jiangsu Provincial Medical Youth Talent (No. QNRC2016043).

## Conflict of Interest

The authors declare that the research was conducted in the absence of any commercial or financial relationships that could be construed as a potential conflict of interest.
